# Neurokinin-1 Receptor Antagonists in Preventing Postoperative Nausea and Vomiting: A Systematic Review and Meta-Analysis

**DOI:** 10.1097/MD.0000000000000762

**Published:** 2015-05-21

**Authors:** Meng Liu, Hao Zhang, Bo-Xiang Du, Feng-Ying Xu, Zui Zou, Bo Sui, Xue-Yin Shi

**Affiliations:** From the Department of Anesthesiology (ML, BD, FX, ZZ, XS), Changzheng Hospital Affiliated to Second Military Medical University, Shanghai, China; Department of Anesthesiology, Second Artillery General Hospital PLA, Beijing, China; Department of Anesthesiology (BD), Second Affiliated Hospital of Nantong University, Nantong, Jiangsu, China; Department of Anesthesiology (XS), Xinhua Hospital Affiliated to Shanghai Jiaotong University School of Medicine, Shanghai, China.

## Abstract

Newly developed neurokinin-1 receptor (NK-1R) antagonists have been recently tried in the prevention of postoperative nausea and vomiting (PONV). This systematic review and meta-analysis was conducted to explore whether NK-1R antagonists were effective in preventing PONV.

The PRISMA statement guidelines were followed. Randomized clinical trials (RCTs) that tested the preventive effects of NK-1R antagonists on PONV were identified by searching EMBASE, CINAHL, PubMed, and the Cochrane Library databases followed by screening. Data extraction was performed using a predefined form and trial quality was assessed using a modified Jadad scale. The primary outcome measure was the incidence of PONV. Meta-analysis was performed for studies using similar interventions. Network meta-analysis (NMA) was conducted to compare the anti-vomiting effects of placebo, ondansetron, and aprepitant at different doses.

Fourteen RCTs were included. Meta-analysis found that 80 mg of aprepitant could reduce the incidences of nausea (3 RCTs with 224 patients, pooled risk ratio (RR) = 0.60, 95% confidence interval (CI) = 0.47 to 0.75), and vomiting (3 RCTs with 224 patients, pooled RR = 0.13, 95% CI = 0.04 to 0.37) compared with placebo. Neither 40 mg (3 RCTs with 1171 patients, RR = 0.47, 95% CI = 0.37 to 0.60) nor 125 mg (2 RCTs with 1058 patients, RR = 0.32, 95% CI = 0.13 to 0.78) of aprepitant showed superiority over 4 mg of ondansetron in preventing postoperative vomiting. NMA did not find a dose-dependent effect of aprepitant on preventing postoperative vomiting.

Limited data suggested that NK-1R antagonists, especially aprepitant were effective in preventing PONV compared with placebo. More large-sampled high-quality RCTs are needed.

## INTRODUCTION

Postoperative nausea and vomiting (PONV) is commonly seen after major surgery. It is estimated that about 30% of the surgical patients will suffer from PONV during the first postoperative day.^1^ The incidence of PONV can be as high as 70% in patients combined with several risk factors such as the use of inhaled anesthetics or opioids, female sex, nonsmoking, and preexisting motion sickness.^2–4^ PONV is distressing to patients, costly and even affects the postoperative recovery profile.^5^ Moreover, successful prevention of PONV might greatly improve patients’ satisfaction.^6^

Several kinds of antiemetics including serotonin 5-HT_3_ receptor antagonists, dopamine receptor antagonists, histamine H_2_ receptor antagonists, anticholinergic agents, and corticosteroids have been tried, which have showed effects on the prevention of PONV.^1^ These drugs mainly act by interfering with neurotransmitter receptors signaling in the central nervous system and gastrointestinal tract except corticosteroids. However, none of the aforementioned antiemetics is universally effective and efficient enough in controlling PONV. In some cases, although several kinds of drugs were provided, they still experience PONV.^7^ Thereafter, more powerful antiemetics are still needed to further reduce the development of PONV.

Neurokinin-1 receptor (NK-1R) is widely expressed in human gastrointestinal vagal afferents and brain areas that are involved in the vomiting reflex such as the nucleus of solitary tract (NST).^8^ Substance P, the natural ligand of NK-1R, was found to be able to trigger NK-1R signaling, thereby causing nausea and vomiting.^9,10^ NK-1R antagonists are believed to provide antiemetic activity mainly by suppressing neuron activities at NST, the central regulator of visceral function.^11^ Several selective NK-1R antagonists have been developed for the prevention and control of nausea and vomiting including aprepitant, fosaprepitant, casopitant, rolapitant, and others. Aprepitant, a highly selective NK-1R antagonist with 9 to 14-hour half-life time, has been approved by FDA for the management of PONV, whereas other ones like rolapitant and casopitant are still under clinical observation. Moreover, NK-1R antagonists have shown great antiemetic activities against chemotherapy-induced nausea and vomiting (CINV), which shared similar traits with PONV.^12^ These results encouraged the prophylactic use of NK-1R antagonists to avoid PONV.^13–15^ However, the clinical effects of NK-1R antagonists on PONV prevention remain inconclusive. To explore whether NK-1R antagonists are effective in preventing PONV, the current systematic review and meta-analysis is performed.

## METHODS

This systematic review and meta-analysis was conducted following the guidelines of PRISMA statement.^16,17^ Ethical approval of our study was not necessary, as this systematic review and meta-analysis did not involve patients.

### Search Strategy

We conducted a literature search of electronic EMBASE, CINAHL, PubMed, and the Cochrane Library databases for articles published before March 31, 2014. The search strategy consisted of a combination of the following free texts and MeSH terms: NK1 receptor antagonists (including neurokinin, NK1, NK1R, NK-1, NK-1R, aprepitant, fosaprepitant, casopitant, rolapitant, ezlopitant, netupitant, CP122721, or vestipitant), postoperative (including surger∗, operation, postoperative, or surgical), and vomiting (including vomit∗, nausea, queasiness, naupathia, retch∗, emesis, or emeses). No language or region restriction was applied. Reference lists of the identified articles were further checked for potential relevant publications.

### Study Selection

Two authors (M.L. and B.D.) independently read the titles and abstracts of the 471 articles returned from the initial search. Articles that were clearly irrelevant according to our predefined inclusion and exclusion criteria were excluded at this phase. Completed studies that met all the following criteria were considered eligible for inclusion in the systematic review and meta-analysis: randomized clinical trials (RCTs) assessing interventions to prevent PONV; participants at least 18 years old, with an American society of Anesthesiologists (ASA) physical status of I to III; and trials comparing the antiemetic effects of NK-1R antagonists with that of other drugs or placebo. Research articles were excluded if they recruited patients with nausea and vomiting before surgery or prophylactic drug administration; were trial protocols or unfinished studies; included nonsurgical patients groups; or enrolled patients with intraoperative chemotherapy. Disagreements on inclusion were resolved by further discussion with a third investigator (X.S.).

### Data Extraction

The primary outcome measure was the incidence of nausea and vomiting. The secondary outcome was the rate of complete response (CR, defined as the absence of vomiting and no need of any rescue antiemetics) and the time to first vomiting (hour). Incidences of using rescue drugs and adverse events were also analyzed if possible. The time point used for data syntheses and comparisons was 24 hours after surgery at which the outcome measures were most frequently reported in the included studies. For a single study, we described all the related outcomes according to the time points listed in the article.

Two authors (M.L. and H.Z.) independently extracted data of all identified trials using a predesigned data collection form. Disagreements were resolved by the third author consultation (X.S.). The following characteristics were collected: primary author, publication year, trial type (single-centered or multicenter trial), participant characteristics (age, sex, and number), types of surgery, anesthesia methods, analgesics and anesthetics, antiemetic prophylaxis (drugs and dosages), the incidence of PONV, the incidence of CR, the time to first vomiting (hour), the percent of using rescue antiemetics, and antiemetics-related adverse events. Dichotomous data were converted into incidences for data syntheses and continuous data were recorded using mean and standard deviation (SD). When incomplete data were encountered, we attempted to contact the authors for details. When no response or no detailed data was provided, we arbitrarily deemed their results as uncertain and ruled out the study for data synthesis.

### Trial Quality Assessment

Two authors (F.X. and Z.Z.) independently read the full texts of included articles and assessed their validity using a modified Jadad scale that we previously described.^18^ The scale evaluated the study quality using the following indicators: randomization, allocation concealment, intervention blinding, withdrawal or dropouts, and intention-to-treat (ITT) analysis. For each indicator, except ITT analysis, 1 point was given when the study used proper methods, and another 1 point was given if the study described them adequately. Otherwise, no point was given. As we selected only randomized trials for analysis, the possible minimal score of an included trial was 1 and the maximum was 8. Studies were not excluded or weighted based on the quality scores in the meta-analysis.

### Data Analysis

Meta-analysis was performed when ≥2 studies using similar interventions were identified. Two control groups were used in our study, placebo and ondansetron, with data analyzed separately. If one study additionally used some NK-1R antagonist to prevent PONV in the intervention group besides routine antiemetics, we arbitrarily classified this kind of studies as studies comparing the antiemetic effect of NK-1R antagonist to that of placebo. As there was no valid method to conduct dose conversion among different NK-1R antagonists (aprepitant, fosaprepitant, casopitant, ezlopitant, netupitant, CP122721, and vestipitant), we performed data syntheses for each drug at every single dose. If relevant data could not be analyzed quantitatively, we reported the results of each study qualitatively with the corresponding *P* values.

RevMan Version 5.2 software (Cochrane Collaboration) was used for data syntheses. Statistical heterogeneity was assessed with a standard χ^2^ and *I*^2^ statistic. Significant heterogeneity was considered existent at χ^2^*P* < 0.10 or *I*^2^ >50% (2-tailed). A fixed-effects parametric approach weighted with the inverse variance was performed when no significant heterogeneity was found. Otherwise, a random-effects model was taken. For dichotomous outcome measures, both pooled risk ratio (RR) and pooled incidence with 95% confidence intervals (CIs) were calculated. For continuous data, standard mean difference (SMD) was used. Publication bias was assessed by visually inspecting funnel plot and using Begg test if needed. For all the analyses, a *P* value of less than 0.05 (2-tailed) was considered statistically significant.

A Bayesian random effects model for multiple treatment comparison was constructed to compare the anti-vomiting effects of aprepitant at different doses.^19^ The network meta-analysis (NMA) was performed by calling WinBUGS 1.4.3 software (MRC Biostatistic Unit, Cambridge, UK) through the R statistical software using the R2WinBUGS package (R Foundation for Statistical Computing, Vienna, Austria). We used Markov chain Monte Carto method in WinBUGS, running 3 chains with different starting values (see Supplemental Digital Content, which describes the R codes in detail). Odds ratio (OR) with 95% CI was presented as summary statistics, and a significant difference was deemed existent when 95% CI of the OR did not include 1.

## RESULTS

### Study Selection

**FIGURE 1:**
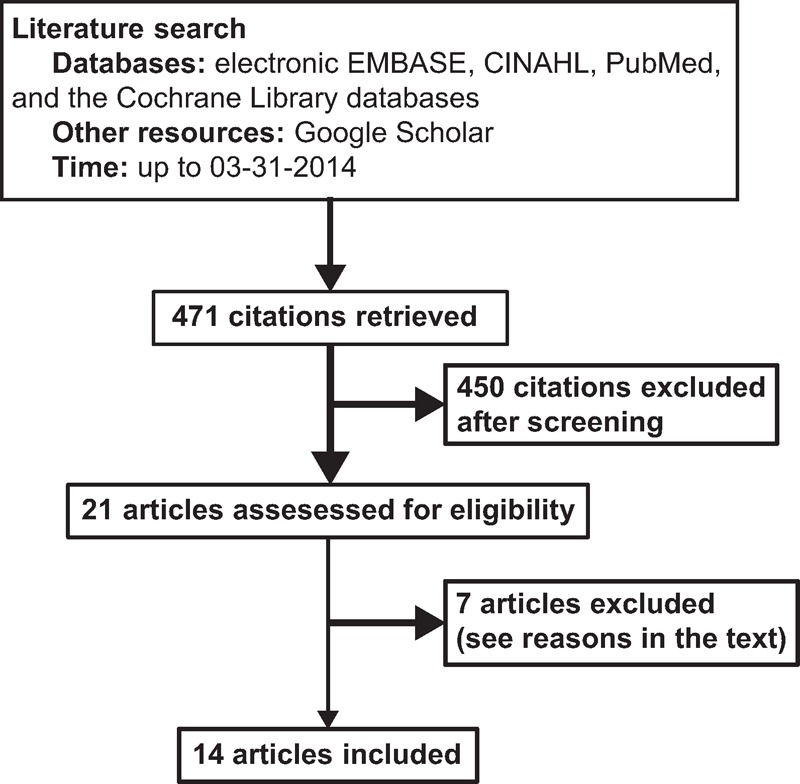
Flow chart of identification, review, and selection of the studies.

The primary search yielded 471 articles. After abstract screening, 21 studies^8,13–15,20–36^ that potentially met the inclusion criteria were identified. The full-text publications of these studies were examined at detail, and 7 trials were further excluded: 2 studies were not RCTs^30,31^; one study described ongoing trials^32^; one study used NK-1R antagonists for patients who already developed nausea or vomiting^36^; one study investigated the efficacy of NK-1R antagonists on postdischarge nausea and vomiting^33^; and 2 studies compared NK-1R antagonist alone to that combined with additional antiemetics.^34,35^ We finally included 14 RCTs^8,13–15,20–29^ in this systematic review and meta-analysis (Figure 1).

### Study Characteristics

The characteristics and main outcomes of included 14 studies^8,13–15,20–29^ were listed in Tables 1 and Tables 2   , respectively. These studies, consisting of 5 multicenter studies^14,20–23^ and 9 single-centered studies,^8,13,15,24–29^ recruited from 60^26^ to 922^20^ patients. The earliest study was published in 2000 by Gesztesi et al.^15^ Thirteen studies were described in English with 1 in Spanish.^26^ The surgery types included otorhinolaryngological,^13^ plastic,^27^ gynecological,^8,15,21,25,28^ abdominal surgeries,^14,20,21,23,26,29^ and craniotomy.^24^ All surgeries were performed under general anesthesia using volatile anesthetics that included sevoflurane, isoflurane, desflurane, or N_2_O (Table 1). The efficacy of aprepitant was tried in ten trials^8,13,14,20,24–29^ with the dosage ranging from 40 to 125 mg. Two studies^21,22^ tested the antiemetic role of different doses of casopitant. The antiemetic efficacy of different dosages of rolapitant^23^ and CP122721^15^ was tried respectively in the rest 2 trials.

**TABLE 1:**
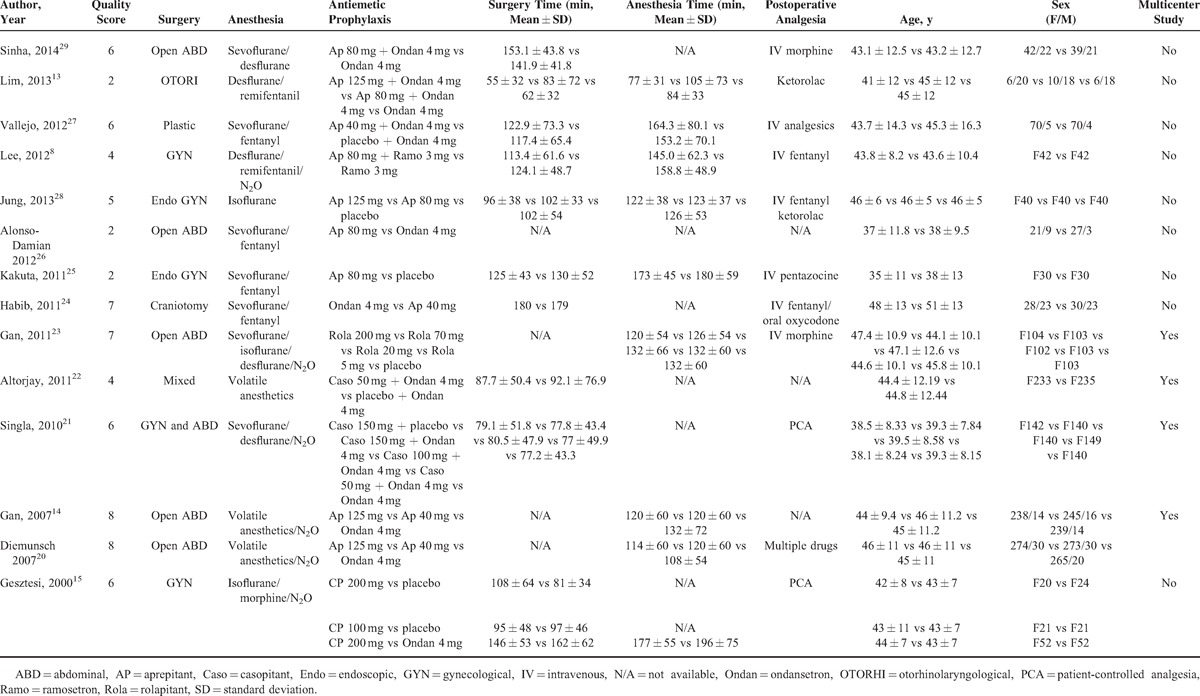
Characteristics of Included Studies

**TABLE 2 (Continued):**
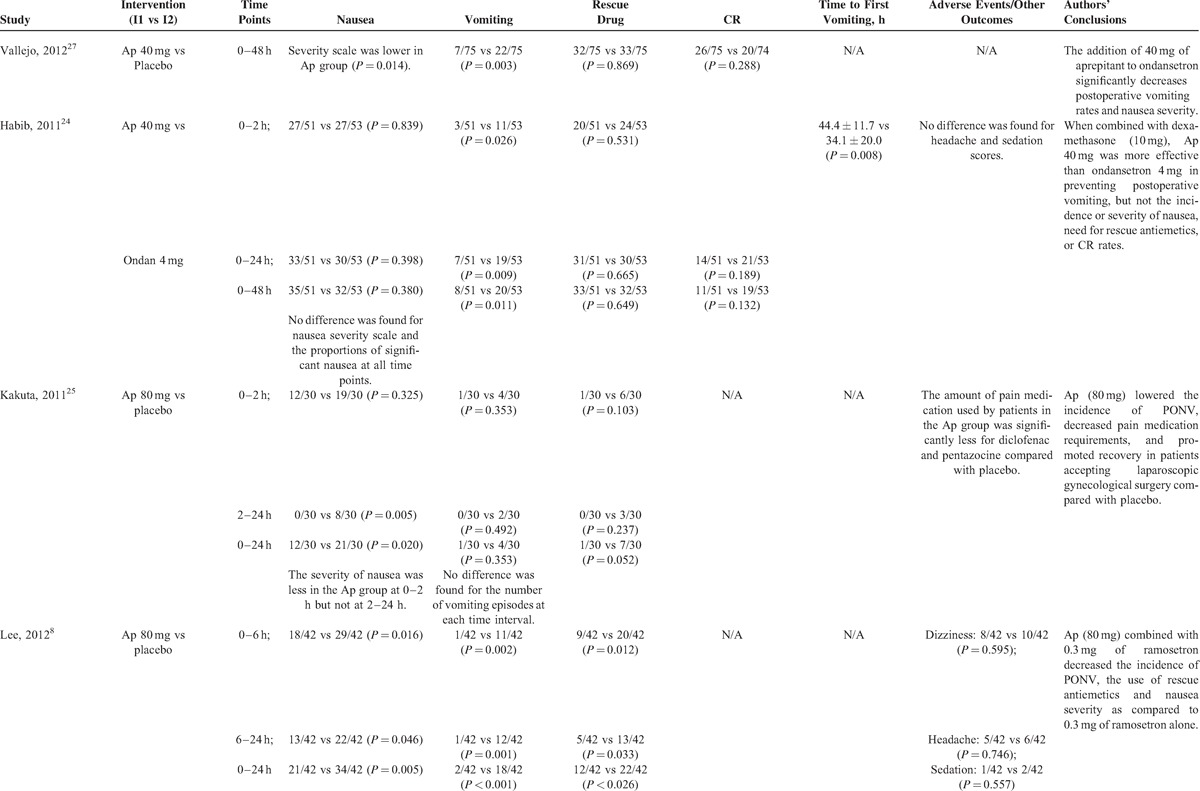
Summarized Outcomes of the Included Studies

**TABLE 2 (Continued):**
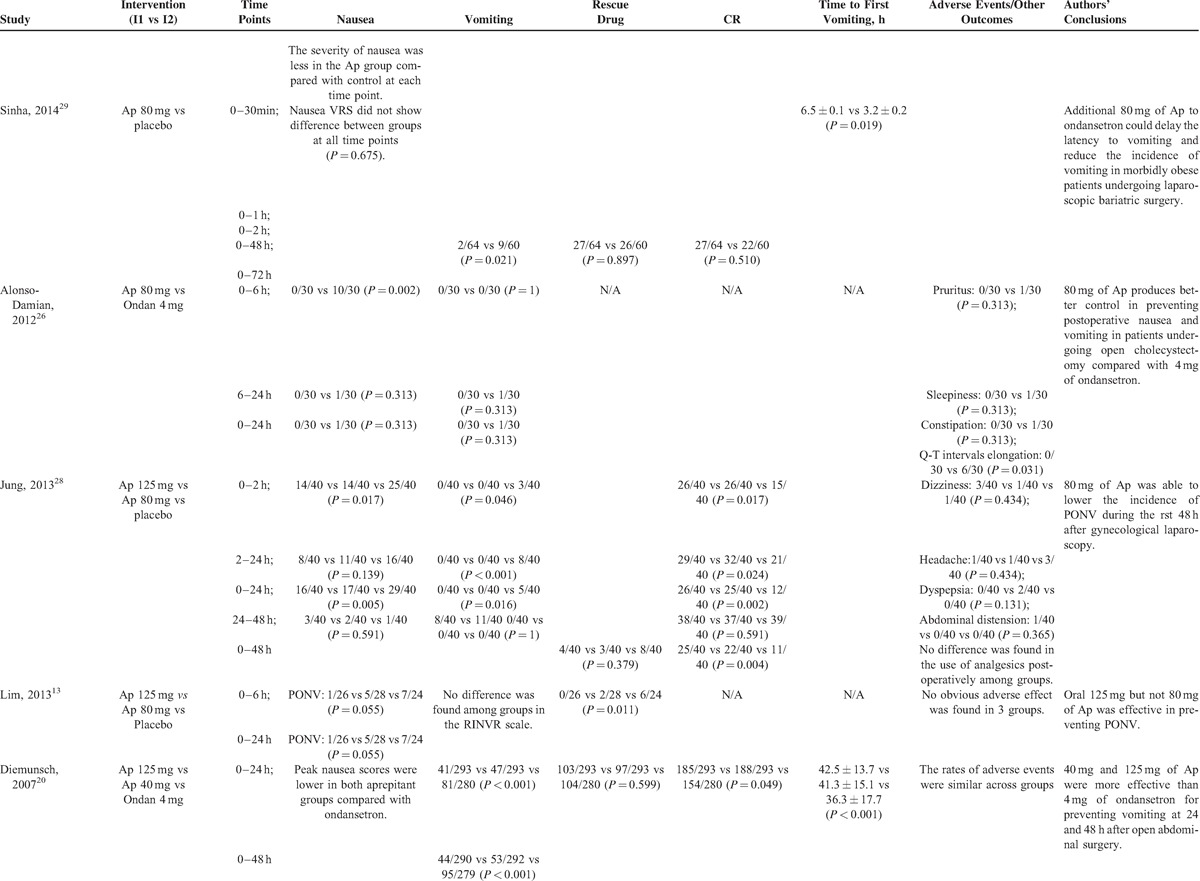
Summarized Outcomes of the Included Studies

**TABLE 2 (Continued):**
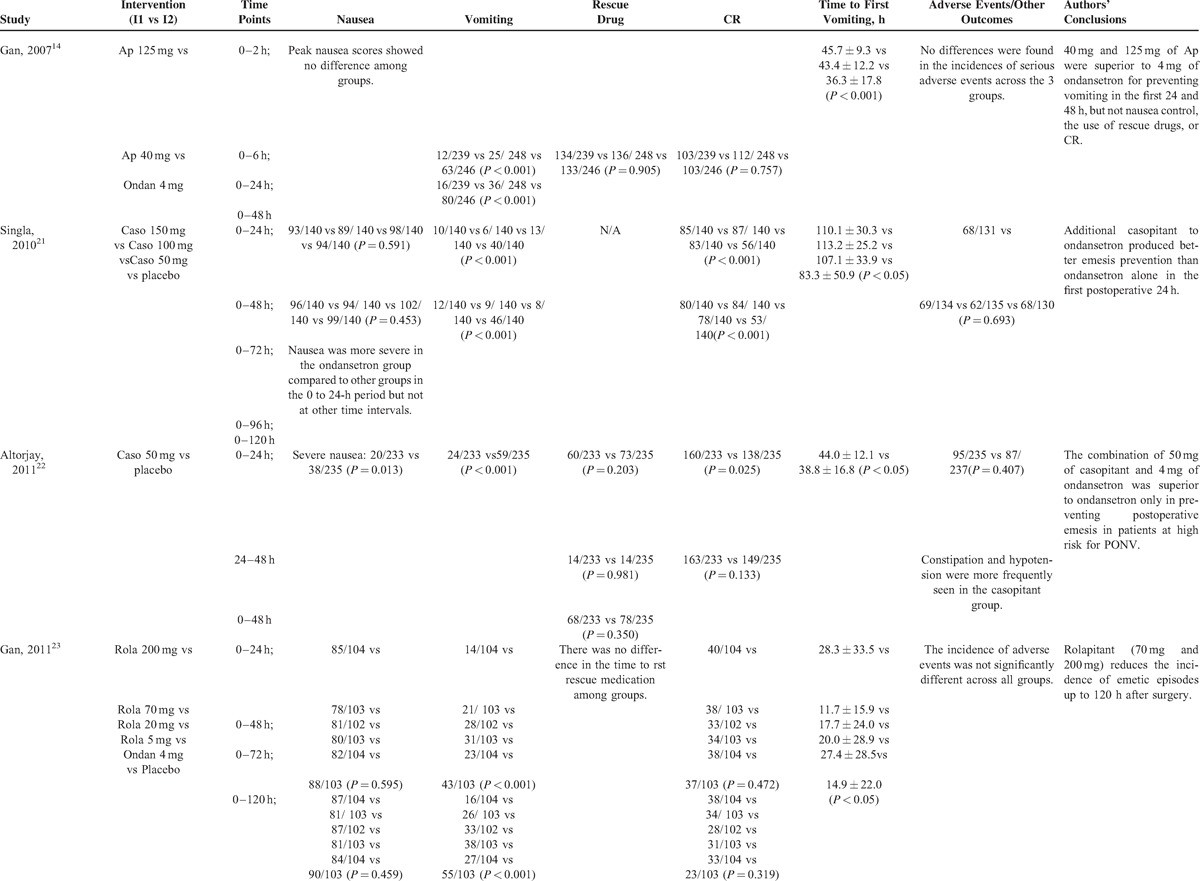
Summarized Outcomes of the Included Studies

**TABLE 2 (Continued):**
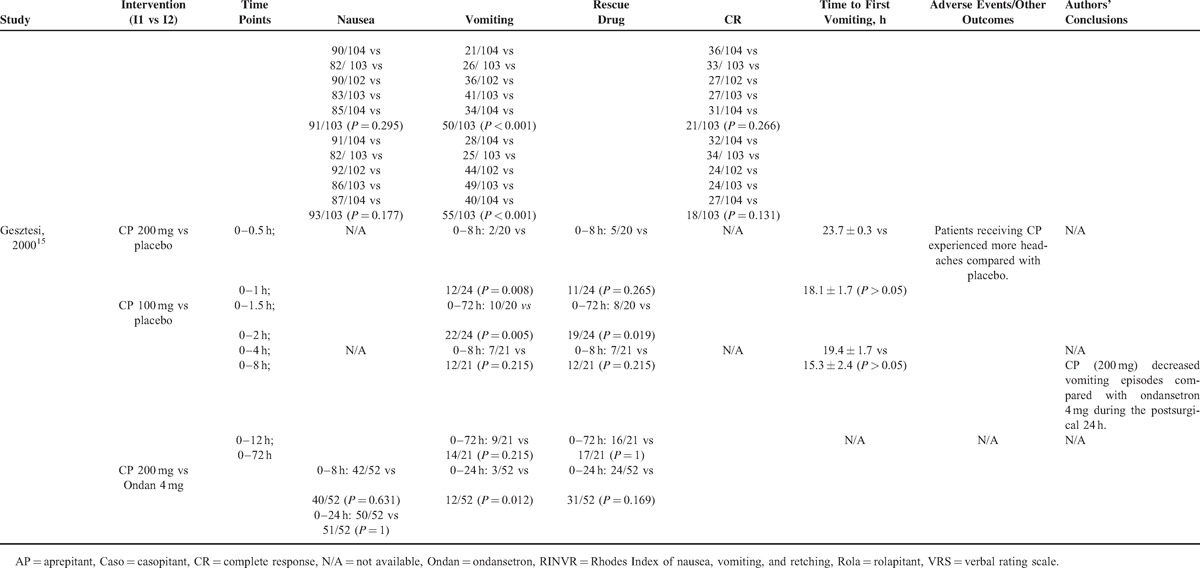
Summarized Outcomes of the Included Studies

### Quality Scores of Included Studies

The scores of included studies were shown in Table 3. The average score was 5.43 with a standard deviation (SD) of 2.03. A score <3 was found in 2 studies.^13,25^ Two studies got a full score of 8.^14,20^

**TABLE 3:**
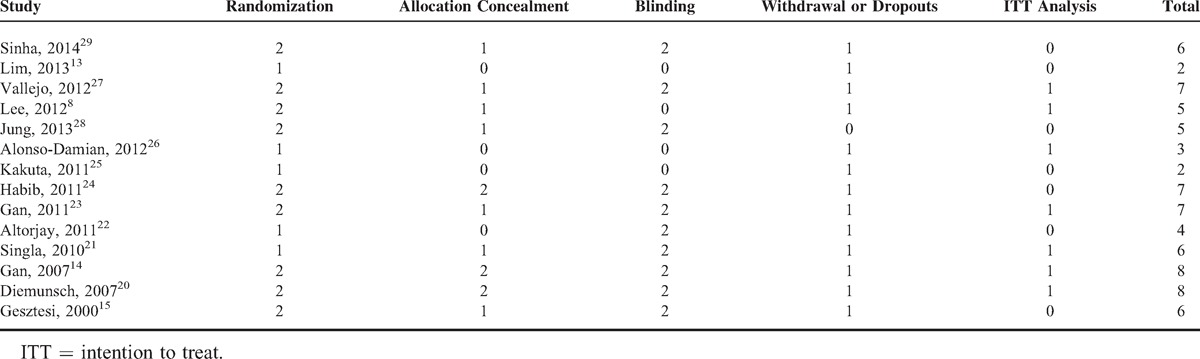
Quality Review of Included Studies

### Quantitative Review and Meta-Analysis

#### Primary Outcomes

##### Incidence of Nausea

**FIGURE 2:**
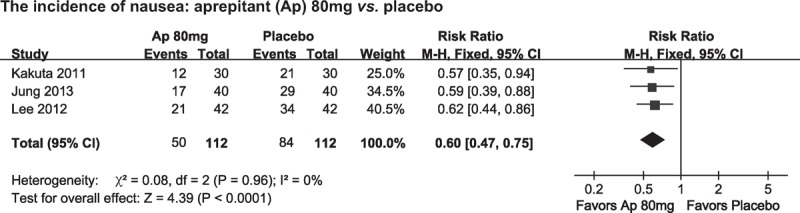
Summarized risk ratios (RRs) for the incidences of nausea.

Of the 8 trials^8,15,21,23–26,28^ reporting the incidence of nausea, 3 studies^8,25,28^ with 224 patients tested the effects of 80 mg of aprepitant versus placebo on preventing postoperative nausea. Meta-analysis using the fixed-effects model showed that prophylactic aprepitant (80 mg) was effective in lowering the incidence of nausea compared with placebo (*P *< 0.001, Figure 2). The pooled incidence of nausea was 45.2% (95% CI = 36.2 to 56.5) in patients receiving 80 mg of aprepitant and was 76.1% (95% CI = 67.8 to 85.4) in patients taking placebo. Jung et al^28^ further showed that 125 mg of aprepitant was also effective in preventing nausea compared with placebo. There was no difference in the incidence of nausea between 2 doses of aprepitant (35% vs 35%; 80 vs 125 mg of aprepitant).^28^

The comparative effects of 80 mg aprepitant and 4 mg ondansetron in preventing postoperative nausea were tested by Alonso-Damian et al.^26^ Their study found that patients taking aprepitant had less nausea on arrival in the recovery room (3.3% vs 53.3%; *P *< 0.001) and 6 hours after surgery (none vs 33.3%; *P *= 0.002) compared with patients taking ondansetron. Moreover, the 2 groups showed similar incidences of nausea during the time intervals of 6 to 24 hours after surgery (0/30 vs 1/30; aprepitant vs ondansetron; *P *= 0.313). However, this study was low in trial quality with a modified Jadad score of 3, and recruited only 30 patients per group.^26^

When aprepitant was taken at a lower dose (40 mg), Habib et al^24^ did not find a significant difference in the incidence of nausea, the proportions of significant nausea or nausea scores between aprepitant and ondansetron (4 mg) at all 3 time points (0–2; 0–24; and 0–48 hours). Both groups of patients received 10 mg dexamethasone after the induction of general anesthesia. Based on their reported incidences of nausea, 708 patients per group were needed to get a significant difference in the incidence of nausea at 48 hours after surgery.

Singla et al^21^ reported that all 3 doses of casopitant (50, 100, or 150 mg) failed to decrease the incidence of nausea compared with placebo. Gan et al^23^ found no difference among 20, 70, and 200 mg of rolapitant and placebo in reducing the occurrence of postoperative nausea.

##### Incidence of Vomiting

**FIGURE 3:**
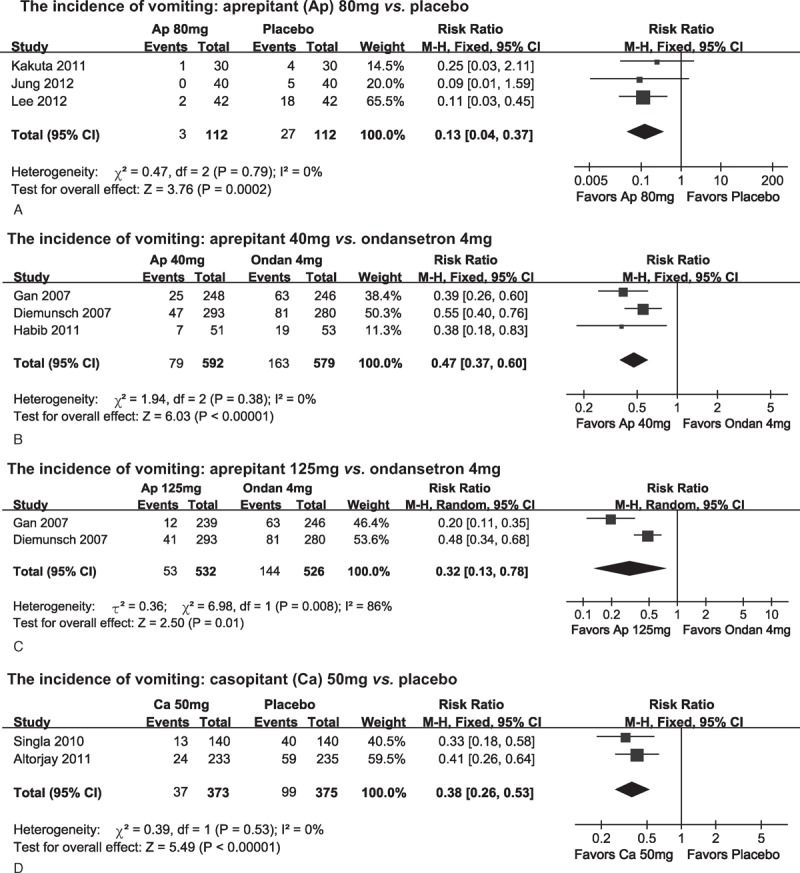
Summarized risk ratios (RRs) for the incidence of vomiting. (A) 80 mg of aprepitant (Ap) vs placebo (placebo). (B) 40 mg of aprepitant vs 4 mg of ondansetron (Ondan). (C) 125 mg of aprepitant vs 4 mg of ondansetron. (D) 50 mg of casopitant (Caso) vs placebo.

Thirteen of the included 14 studies^8,13,14,20–29^ reported the incidence of vomiting. The reported incidences ranged from 15%^29^ to 50%^8^ in these studies. When the anti-vomiting role of aprepitant was compared with placebo, meta-analysis of 3 studies recruiting 224 patients^8,25,28^ found that 80 mg of aprepitant could lower the proportions of patients suffering from postoperative vomiting compared with placebo (*P *< 0.001, Figure 3A). The pooled incidence was 3.8% (95% CI = 1.1 to 12.8) for 80 mg of aprepitant and was 21.1% (95% CI = 8.2 to 54.0) for placebo. Sinha et al^29^ reported that the incidence of vomiting at 72 hours after surgery was significantly lower in patients receiving additional 80 mg of aprepitant (3.1% vs 15%; *P *= 0.021).

For other doses of aprepitant tested, Vallejo et al^27^ reported that 40 mg of aprepitant was more effective in preventing vomiting than placebo (RR = 0.31; 95% CI = 0.14 to 0.69; *P *= 0.003). Jung et al^28^ found that none of the 40 patients receiving 125 mg of aprepitant developed vomiting while 11 of the 40 patients receiving placebo were diagnosed with postoperative vomiting (*P *= 0.03).

Three studies^14,20,24^ with 1171 patients compared the roles of 40 mg aprepitant and 4 mg ondansetron in reducing postoperative vomiting. Meta-analysis using the fixed-effects model revealed that 40 mg of aprepitant was more effective than 4 mg of ondansetron in preventing vomiting (*P *< 0.001, Figure 3B). The pooled incidence was 13.3% (95% CI = 9.5 to 18.4) for 40 mg of aprepitant and was 28.4% (95% CI = 24.6 to 32.9) for 4 mg of ondansetron.

Alonso-Damian et al^26^ did not find a superior role of 80 mg of aprepitant in preventing postoperative vomiting in patients accepting open abdominal surgery compared with 4 mg of ondansetron. The study recruited only 30 patients for each group and the reported incidences of vomiting were low (0/30 vs 1/30; 80 mg aprepitant vs 4 mg ondansetron). Based on their reported incidences of vomiting, 311 patients per group were needed to get a significant difference.

Meta-analysis of the 2 studies^14,20^ recruiting 1058 patients found that 125 mg of aprepitant was more effective in reducing the incidence of vomiting compared with 4 mg of ondansetron (*P *= 0.01, Figure 3C). The pooled incidence was 8.7% (95% CI = 3.2 to 23.6) for 125 mg of aprepitant and was 27.5% (95% CI = 23.8 to 31.7) for ondansetron.

In terms of other NK-1R antagonists, synthesized data from 2 studies^21,22^ suggested that 50 mg of casopitant could further decrease the incidences of vomiting by 65.1% compared with placebo (pooled incidences, 9.9% vs 25.5%) (Figure 3D).

Gan et al^23^ found that patients assigned to 20, 70, and 200 mg of rolapitant had lower incidences of emesis (27%, 20%, and 13%, respectively) compared with patients taking placebo (42%). There was a linear relationship between the incidence of vomiting and the dose of rolapitant. Gesztesi et al^15^ performed a dose-ranging and interaction study of CP122721 to test its antiemetic effects. In their dose-ranging study, 10% (2/20) of the patients that received 200 mg of CP122721 experienced vomiting within the first 8 hours after surgery in comparison with that 50% (12/24) of the patients in the placebo group were found to experience vomiting (*P *= 0.008). In the interaction study, the effects of 4 mg of ondansetron and 200 mg of CP122721 alone, and their combinational effects on preventing PONV were tested. The incidences of PONV within the first 2-hour post-surgical period were 6% in patients treated with 200 mg of CP122721, 17% in patients taking 4 mg of ondansetron and 2% in patients receiving both CP122721 and ondansetron (*P *< 0.05).

#### Secondary Outcomes

##### Use of Rescue Drugs

A total of 11 studies^8,13–15,20,22,24,25,27–29^ reported the incidence of using rescue drugs in treating post-surgical nausea and vomiting. Meta-analysis of 2 studies^8,25^ recruiting 144 patients showed that patients receiving 80 mg of aprepitant were less likely to take rescue drugs compared with those taking placebo (pooled RR = 0.45; 95% CI = 0.26 to 0.77; *P *= 0.004). Vallejo et al^27^ tested the comparative effects of 40 mg of aprepitant with placebo on reducing the use of rescue drugs and did not find a significant difference between 40 mg of aprepitant and placebo.

When aprepitant was compared with 4 mg of ondansetron,^14,20,24^ no significant difference was found between 40 mg of aprepitant and ondansetron (n = 3, pooled RR = 0.97; 95% CI = 0.86 to 1.10; *P *= 0.65); or between 125 mg of aprepitant and ondansetron (n = 2; pooled RR = 1; 95% CI = 0.87 to 1.14; *P *= 0.96) in the incidences of using rescue drugs.^14,20^

In the trial by Gesztesi et al,^15^ 200 mg but not 100 mg of CP122721 was found to be effective in decreasing the use of rescue drugs compared with placebo during the first 72 hours after surgery (*P *= 0.019 for 200 mg CP122721 vs placebo; and *P *= 1 for 100 mg CP122721 vs placebo). Moreover, there was no difference in the incidence of using rescue drugs between 200 mg of CP122721 and 4 mg of ondansetron (*P *= 0.169). Based on their reported incidences, 290 patients per group were needed for 200 mg of CP122721 and 4 mg of ondansetron to get a significant difference.

##### Complete Response

There were 8 studies^14,20–24,27–29^ that reported the CR values of NK-1R antagonists. The effects of aprepitant (40 mg) against ondansetron (4 mg) were tested in 3 studies.^14,20,24^ Meta-analysis using the random-effect model found no significant difference between 40 mg of aprepitant and ondansetron (pooled RR = 1.08; 95% CI = 0.91 to 1.29; *P *= 0.36). The pooled incidence was 46.3% (95% CI = 32.5 to 65.7) for patients receiving 40 mg of aprepitant and 46.3% (95% CI = 36.9 to 58.0) for patients taking 4 mg of ondansetron. There was no difference in CR rates between 125 mg of aprepitant and 4 mg of ondansetron (N = 2; pooled RR = 1.10, 95% CI = 0.98 to 1.24; *P *= 0.1).^14,20^

When the effects of NK-1R antagonists on CR rates were compared with placebo, Vallejo et al^27^ found no beneficial effect of 40 mg of aprepitant (37.3% vs 26.7%; *P *= 0.288). Based on these incidences, 405 patients per group were needed to get a difference between 40 mg of aprepitant and placebo whereas study by Vallejo et al^27^ recruited only 75 patients per group.

When additional 50 mg of casopitant was used besides 4 mg of ondansetron, meta-analysis from 2 studies^21,22^ recruiting 748 patients supported that patients receiving additional casopitant were more likely to be diagnosed with CR than those taking placebo (pooled RR = 1.26; 95% CI = 1.12 to 1.42; *P *< 0.001). In addition, Gan et al^23^ found that there was an increase in CR rates in the rolapitant group (70 and 200 mg) compared with that in the placebo group during the postoperative 48-to-72-hour period.

##### Time to First Vomiting Episode

Eight studies^14,15,20–24,29^ reported the time to first vomiting episode after surgery. We identified 3 studies^14,20,24^ with 1171 patients that compared the effects of 40 mg aprepitant with 4 mg of ondansetron on the time to first vomiting. Meta-analysis using the fixed effects model showed that 40 mg aprepitant could delay the time to first vomiting compared with ondansetron (pooled SMD = 0.40; 95% CI = 0.28 to 0.51; *P *< 0.001).

Another 2 studies^14,^^ 20^ recruiting 1058 patients evaluated the effects of 125 mg of aprepitant and 4 mg of ondansetron on the time to first vomiting. The synthesized results using a random-effect model suggested that 125 mg of aprepitant was more effective in delaying the vomiting latency compared with 4 mg of ondansetron (pooled SMD = 0.52; 95% CI = 0.26 to 0.78; *P *< 0.001).

Altorjay et al^22^ found a postponement of first vomiting by 50 mg of casopitant compared with placebo and the relative hazard ratio for the risk of emesis was 0.414 (95% CI = 0.265 to 0.646). Singla et al^21^ reported a superior effect of 50 mg of casopitant on the delay of time to the first vomiting. We did not synthesize the data because of huge heterogeneity.

Gan et al^23^ found that the median time to first vomiting episode was longer in patients receiving 200 mg of rolapitant and shorter in patients receiving 70 mg of rolapitant compared with patients receiving placebo. Gesztesi et al^15^ reported that 200 mg of CP122721 could delay the onset of emesis compared with placebo.

### NMA for the Incidences of Vomiting

**FIGURE 4:**
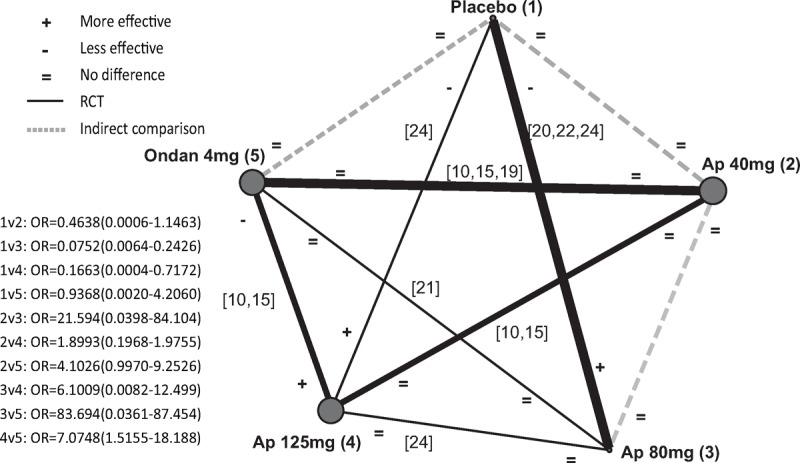
Network meta-analysis for the incidences of vomiting among placebo, ondansetron, and different doses of aprepitant. Solid lines connecting 2 treatments denote direct comparisons and dotted lines denote indirect comparisons. A thicker line means more studies included. A bigger dot denotes a larger population. Ap = aprepitant, Ondan = ondansetron, OR = odds ratio.

NMA was performed to further compare the effects of placebo, ondansetron, and different doses of aprepitant on postoperative vomiting occurrences. A total of 7 studies^8,14,20,24–26,28^ were included in the present NMA. As shown in Figure 4, higher doses of aprepitant (80 and 120 mg) but not 40 mg of aprepitant was effective in preventing post-surgical vomiting compared with placebo. No significant difference was found among different doses of aprepitant. Meanwhile, 125 mg of aprepitant bears significant superiority in the prevention of vomiting in comparison with 4 mg of ondansetron.

## DISCUSSION

Our current systematic review and meta-analysis supported the following findings. Firstly, drugs and dosages of NK-1R antagonists used for preventing PONV are still being explored and differ a lot among the 14 identified trials. Secondly, based on the synthesized and individual data as well as the trial quality, higher doses of aprepitant (80 and 125 mg), casopitant (100 and 150 mg), rolapitant (20, 70, and 200 mg), and CP122721 (200 mg) were effective in preventing PONV compared with placebo (Table 4). However, the effects of NK-1R antagonists against ondansetron in reducing PONV occurrence were uncertain. Last but not the least, available data did not find a dose-related effect of aprepitant in preventing PONV. More large high-quality trials are needed to clarify this question.

**TABLE 4:**
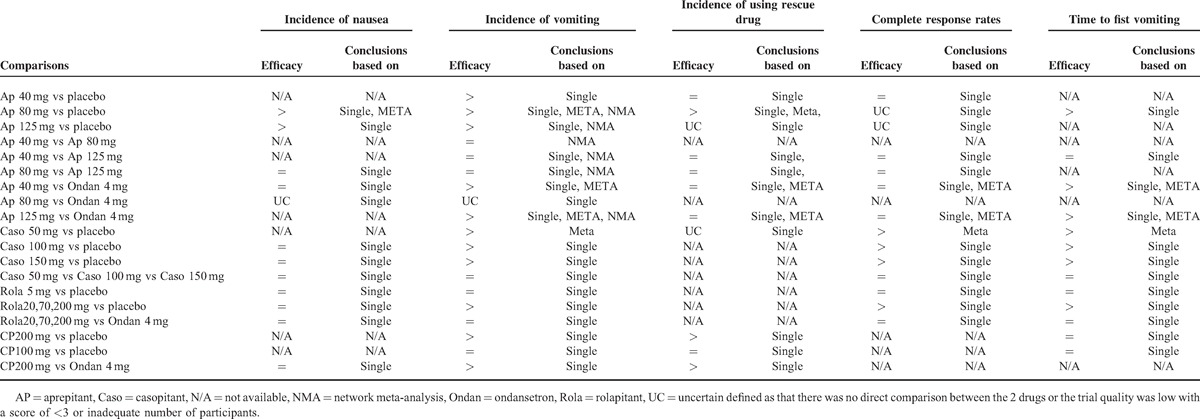
Summarized Efficacy Comparisons Among Placebo, Ondansetron, and Neurokinin-1 Receptor Antagonists

As the first NK-1R antiemetic approved by the FDA, aprepitant was the mostly tested agent in our identified trials.^8,13,14,20,24–29^ The current meta-analysis found that all dosages of aprepitant (40, 80, and 125 mg) were effective in reducing the incidence of postoperative vomiting but not the rates of nausea. The dissociative effect on nausea and vomiting was also seen in casopitant, rolapitant, and CP122721 (Table 4). These results supported the hypothesis that nausea and vomiting were 2 biologically different phenomena that occur due to common but differentiated etiologies.^37,38^ Moreover, our current findings were similar to previous reports.^39,40^ Albany et al^40^ found that when combined with a 5-HT_3_ receptor antagonist and dexamethasone, aprepitant could more effectively suppress nausea but not vomiting in tumor patients receiving cisplatin combination chemotherapy. Therefore, NK-1R antagonists might be better used at a multimodal approach to efficiently control PONV.^41^

Previous studies have found an antiemetic role of NK-1R antagonists in patients receiving highly emetogenic chemotherapy (HEC),^42^ as well as moderately emetogenic chemotherapy (MEC).^43^ In addition, the meta-analysis by dos Santos et al^12^ showed that NK-1R antagonists improved the control of CINV during the acute (0–24 hours), delayed (24–120 hours), and overall phases in patients who received HEC and MEC. Similarly, our meta-analysis found that NK-1R antagonists especially aprepitant were effective in controlling PONV in patients undergoing different kinds of surgery. These results suggested that NK-1R antagonists with a long duration had a strong potency in controlling different kinds of nausea and vomiting. As the studies included in our meta-analysis did not state whether the surgeries were tumor-related, we could not exclude an effect of tumor on the antiemetic role of NK-1R antagonists. However, NK-1R antagonists have shown great antiemetic benefits in patients with breast cancer,^44^ lung cancer,^45^ head and neck cancer,^46^ and others. The efficacy of NK-1R antagonists were also confirmed in the study by Rapoport et al^43^ who recruited patients with different tumor types, including breast cancer, lung cancer, colon cancer, and ovarian cancer. Thus, we thought it was reasonable to conclude that NK-1R antagonists, especially aprepitant, helped preventing PONV in patients undergoing surgery with general anesthesia.

Our NMA including both direct and indirect data did not find a difference in preventing vomiting among different dosages of aprepitant (Figure 4). This finding suggested that aprepitant was a powerful antiemetic drug and low-to-moderate dose of aprepitant might be sufficient to control PONV with minimal adverse effects. However, we suggested that the finding should be taken with caution. As there were only 7 studies included in the NMA, a great bias was likely to be existent.^47,48^ Furthermore, the NMA results suggested that 80 and 125 mg but not 40 mg of aprepitant were superior to ondansetron in controlling postoperative vomiting. Considering these data, more clinical trials with high quality were needed to test the most appropriate dosage of NK-1R antagonists in preventing PONV.

Another conclusion that could be drawn from our study was that NK-1R antagonists, especially rolapitant and casopitant could delay the time to first vomiting episode, compared with ondansetron. This might be mainly due to their longer acting time compared with ondansetron.^49^ One alternative explanation was the different acting mechanisms of NK-1R and 5-HT_3_ receptor antagonists, as studies had suggested that vomiting at early phase and late phase might be caused by different drugs used in the perioperative settings.^1,41^

Twelve of the 14 studies reported adverse events or dissatisfactions of patients.^8,13–15,20–26,28^ We did not synthesize the data because of insufficient reports and huge heterogeneity among studies. Generally, the most frequently reported adverse events of NK-1R antagonists were headache (2.5%–22%), dizziness (7.5%–19%), and constipation (7.2%–9%). There were also drug-specific adverse events. For example, hypotension was reported in a larger proportion of cases who received casopitant than that taking placebo,^21,22^ and more episodes of headache were found in patients taking CP122721 than that with placebo.^15^ Studies have reported other adverse effects of NK-1R antagonists. For instance, dos Santos et al^12^ proposed that NK-1R antagonists might impair natural defenses and increased the opportunity of severe infection when used for the control of CINV. Whether this adverse effect would exist in perioperative patients was unknown and none of our included studies mentioned that problem.

This systematic review and meta-analysis had several limitations. First, we included different types of surgeries for a single intervention and this added heterogeneity to our analysis. Second, Apfel et al^50^ described that female sex, a history of motion sickness or PONV, nonsmoker, and the use of opioids after surgery were independent predictive factors for PONV. The studies identified in our review included patients with various levels of susceptibility to PONV. For example, 7 studies include only female patients.^8,15,21–23,25,28^ This might be another source of heterogeneity. Thirdly, some studies were small-sampled single-centered studies or got low quantitative scores for their less methodological rigor compared with large-sampled studies. This might lead to an overestimation of effect sizes in small trials.

Our review raised several questions that needed to be addressed in future studies. Firstly, there were limited data on NK-1R antagonists for preventing PONV and large-sample high-quality studies were in urgent need to confirm our conclusion. Secondly, high-risk and low-risk patients might have different sensitivity to antiemetics. Thus, more data from low-risk patients were needed for future studies. Third, the description of adverse events in our identified studies was relatively simple that might lead to an underestimation of the potential hazards. Given this situation, future RCTs should pay more attention to the side effects of NK-1R antagonists.

In conclusion, our study found that NK-1R antagonists, especially aprepitant, helped preventing PONV in patients undergoing surgery with general anesthesia by decreasing the incidence of nausea and vomiting, and delaying the time to first vomiting. However, more data from high-quality RCTs and a comprehensive evaluation of related adverse events were needed before a recommendation of using NK-1R antagonists to prevent PONV could be made.
